# Fostering Teachers’ Multicultural Competence for Chinese Ethnic Minority Education: An Analysis of Teacher Education Programmes, Syllabuses and Teacher Educator Perceptions

**DOI:** 10.3389/fpsyg.2022.810240

**Published:** 2022-02-24

**Authors:** Wei Wang

**Affiliations:** Research Center for Rural Education, Faculty of Educational Science, Hunan Normal University, Changsha, China

**Keywords:** multicultural competence, ethnic minority education, teacher education programme, syllabus, educational governance, China

## Abstract

The multicultural characteristics of students belonging to ethnic minorities in China pose challenges for teachers. Teacher competence in dealing with culturally diverse students has been extensively discussed in international scholarship and referenced by Chinese researchers, but there is limited empirical research on how teacher education programmes in China respond to this challenge and theoretical discussions. Based on content analysis on teacher education programmes and syllabuses, as well as expert interviews with four teacher educators at two teacher education institutions, this study investigates how the cultivation of multicultural competence is incorporated into teacher education programmes, and the external forces that shape it. Drawing on international scholarship on teachers’ multicultural competence and Cochran-Smith’s framework on external forces influencing multicultural teacher education practices, I argue that the cultivation of teachers’ multicultural competence for their future work in ethnic minority education is, to a great extent, missing from teacher education programmes. Furthermore, what pre-service teachers’ competence covers, and the external forces that influence how teacher education plays out in practice, are influenced and somewhat determined by the large social, economic and political context as well as the agenda for educational reform in China.

## Introduction

When it comes to emotional expression, the students from the Dai [ethnic group] are gentle and, by contrast, the ones from Bulang and Hani are stubborn. As a teacher, I must use differential treatment in my teaching and management strategies. (Teacher Hu, Xishuangbanna, Yunnan, 2017-5-9)

Last year, a teachers’ team from Shanghai came to our school to help us enhance teaching quality. […] It was interesting that the average score of the class taught teach was lower than those our teachers taught in the semester of the final examinations. I know. […] Even if they have rich knowledge and advanced teaching skills, in our area they also need to understand students’ cultural psychological characteristics and change their teaching and management style to adapt to our ethnic minority students. Otherwise, students will dislike and resist these teachers and be unable to concentrate on their studies. (Teacher Liu, Xishuangbanna, Yunnan, 2017-5-17)

These are the voices of two teachers working in a junior secondary school located in Xishuangbanna Dai Autonomous Prefecture in China’s Yunnan Province, where my earlier fieldwork took place. As these teachers’ narratives show, the distinct cultural characteristics of diverse ethnic minority students can pose challenges to teachers.

Building teachers’ competence in coping with diverse students has been discussed by many scholars in the field of multicultural education (e.g., Mushi, 2004; Seeberg and Minick, 2012). Training pre-service teachers to build multicultural competence, including cultural knowledge, multicultural pedagogical knowledge and skills, is perceived as a vital mission of teacher education programmes in the twenty-first century (Gay and Howard, 2000). Drawing on this scholarship (Banks, 1993), since the turn of the millennium a group of Chinese educational researchers has discussed the importance of multicultural competence for teachers who work in multicultural or multi-ethnic areas of China (e.g., Te, 2007; Bai, 2008; Ni, 2014). One central claim is that teacher education programmes in China should take into consideration the demographic situations of ethnic minority areas and enable pre-service teachers to facilitate culturally diverse students through the practice of ethnic minority education. However, by comparison with international scholarship which has a large body of work examining teacher education on multicultural education at a practical level in different countries (Gorski, 2009; Egne, 2014), in Chinese academia empirical studies are, unfortunately, underrepresented (Wang, 2018).

To examine how multicultural competence is reflected in existing teacher education programmes in China, I collected and analysed two syllabuses from teacher education programmes and the perceptions of four teacher educators working at the two institutions from which the syllabuses were drawn. In addition, drawing on the conceptualisation framework for multicultural teacher education developed by Cochran-Smith (2003) which elaborates how external forces shape teacher preparation practices, I will clarify the external forces that influence teacher education programmes in China. In connecting these research aims, I seek to answer the following research questions: (1) To what extent do teacher education programmes for pre-service teachers in China incorporate training to build multicultural competence? (2) What are the underlying external forces that influence the extent to which teacher training fosters competence in providing ethnic minority education?

The contributions of this study are twofold. By examining teacher education syllabuses, the study explores current teacher education programmes and how they build teachers’ multicultural competence, thereby bridging the gap between theoretical discussions and empirical research mentioned above. Secondly, through interviews with teacher educators, this study summarises the external forces that shape teacher education practices in China. Thus, this study can provide references for future academic research and policymaking in reforming teacher education programmes to be inclusive in serving ethnic minority education.

## The Chinese Context: The Demographic Situation in Schools in Ethnic Minority Regions

The People’s Republic of China was established in 1949 by the Chinese Communist Party as a multi-ethnic, multicultural and multilingual state (Lee, 2016). The central government currently recognises 56 ethnic groups: the majority Han and 55 ethnic minority groups. These 55 ethnic minority groups comprise slightly less than 10% of the population, yet only ten countries in the world have populations larger than Chinese ethnic minorities (Postiglione, 2009, p. 501). The reality of Chinese society is multilingual: there is one official language, Standard Chinese (*putong hua*), and more than 80 ethnic minority languages (Wei et al., 2021). In addition, the number of foreign language learners/users within China reached 415.95 million by 2,000 (Kong and Wei, 2019).

Briefly speaking, these 55 ethnic minorities were identified on account of their distinct culture and differences from the majority Han and one another, particularly in terms of customs, languages, religions, and so on (Lee, 2016). “Ethnic minority education” can be envisaged as education for a homogenous classroom consisting of students from a single ethnic minority group. However, in China, the reality is different, to a great extent. Ethnic minorities exist in “an extensive dispersion with localised concentrations” (*dazajü xiaojüjü*), meaning that their populations are mixed into the broader population in provinces and cities. Meanwhile, individual ethnic minority groups are concentrated in smaller administration units such as villages. However, since 2001 a “merged school” (*chedian binxiao*) education policy has been implemented, closing small village schools and concentrating student into centralised schools in urban areas (Cai and Kong, 2014). This initiative aims to solve the problem of imbalanced distribution in education resources and to promote education equality, but in ethnic minority areas the move from village schools serving single ethnic minority groups to multicultural schools in towns and cities has created a demographic situation for ethnic minorities that is, in essence, heterogeneous. This multicultural reality inevitably challenges teachers working in ethnic minority education and increases the need for teacher competence in dealing with culturally diverse students. Thus, developing multicultural competence amongst teachers should be a concern of teacher education programmes.

## Theoretical Framework

The primary focus of this study is to examine how Chinese teacher education programmes foster pre-service teachers’ multicultural competence and the underlying influential factors on such programmes. The study will discuss the extent to which teacher education programmes incorporate pre-service training in multicultural competence. In addition, I will analyse pre-service programmes using the Cochran-Smith (2003) framework for multicultural teacher education, part of which focuses on how teacher education programmes are influenced by external forces.

### Conceptualisation of Teachers’ Multicultural Competence

Awareness of the necessity of teachers’ competence in teaching culturally diverse students has grown in the field of multicultural education, as has scholarship on multicultural education and its related area of teacher education. Within this field there are different concepts, such as multicultural competence (Banks, 2014), cross-cultural competence (McAllister and Irvine, 2000) as well as intercultural competence (Mushi, 2004), but these share notions of teachers’ competence for effectively teaching culturally diverse students and can largely be used interchangeably. For example, Mushi (2004) argues that teachers engaged in a well-planned multicultural classroom should grasp intercultural competence for effective teaching. In the rest of this article, I will use the term multicultural competence for these concepts. To answer the first research question in this study and examine the practices of teacher education programmes in China, I examine the related literature on conceptualising teachers’ multicultural competence.

Multicultural competence is regarded as the essential quality and ability of teachers to effectively engage with diverse students. Many multicultural education researchers, such as Gay and Howard (2000), Villegas and Lucas (2002), Mushi (2004), and Seeberg and Minick (2012) etc., discuss teachers’ multicultural competence in three dimensions:

1. *Awareness*: Multicultural awareness is widely regarded as preceding the other two dimensions (Cherng and Davis, 2019). It requires teachers to have the cultural sensitivity to recognise that students’ ways of thinking and behaving are profoundly influenced by their cultural backgrounds, by demonstrating an acceptance of difference, anti-racist perspectives etc. (Villegas and Lucas, 2002). The teacher should respect and eliminate prejudice against different cultures, developing a positive attitude to facilitate students from culturally diverse backgrounds (Mushi, 2004).
2. *Knowledge:* The teacher should have factual knowledge of minority cultures and cultural diversity to bring them into educational activities, help reduce prejudice, create cross-cultural awareness and avoid stereotyping culturally diverse students. The teacher should also understand the principles and ideology of multicultural teaching and theories in multicultural education (Melnick and Zeichner, 1997; Banks, 2014; Yuan, 2018).
3. *Skills:* As the opposite of cultural deficit (or cultural deprivation) theory which attributes lower academic performance to students’ cultures and recommends compensatory interventions, cultural difference theory recommends that the teacher should have a grasp of culturally relevant content and pedagogical strategies to create classroom interventions that facilitate effective learning for diverse students (Ladson-Billings, 1995; Gay, 2002). Culturally responsive teaching helps teachers respect and care for students’ diverse cultural backgrounds and develop effective skills to translate multicultural knowledge into classroom instruction and curriculum design, making education inclusive for culturally diverse students (Gay, 2018; Abacioglu et al., 2020).

However, these conceptions of teachers’ multicultural competence are not changeless, but evolving and expanding (Grant and Agosto, 2008). For example, critical multiculturalism, which relates to challenging the power relations with a given society, requires teachers to stand in a broader socio-political context to consider their work in schools (Gorski, 2009). Critical multiculturalism shapes an ideology which guides teachers to develop the competence “to confront and engage the world critically and challenge power relations” (Sleeter and McLaren, 1995, p. 7). Therefore, teachers should be conscious of how various biases are produced economically, politically and institutionally, and how students’ identities are shaped in unequal power relationships, and to act accordingly (Kim and Choi, 2020). Critical consciousness enriches the three dimensions of multicultural consciousness described above rather than being an independent strand, and can help teachers develop critical reflexivity toward underlying inequality in curricula, school culture and society to promote education equality (May, 2009; Forrest et al., 2016).

### External Factors Influencing Multicultural Teacher Education

Teacher education, which is responsible for training teachers in multicultural competence, is shaped by many external forces. Cochran-Smith (2003) identifies three such forces.

The first external force, *institutional capacity and mission* relates to how teacher education institutions can implement approaches to teacher preparation and training in the context of the broader mission. Second, *relations with local communities and schools* describes the importance of interacting with the authentic multicultural context in teacher preparation programmes. Finally, *governmental/non-governmental regulation* refers to how teacher education programmes are regulated and influenced by agencies with authority over them. On top of this framework is the *larger context* including the political, social, economic, and historical domains as well as the educational reform agenda which has implications for all three external forces as well as teacher education. This framework is significant to this study as it enables an examination of the external forces that influence the effectiveness of teacher education institutions in cultivating teachers’ multicultural competence in their programmes, as well as the broader reform agenda in China.

**FIGURE 1:**
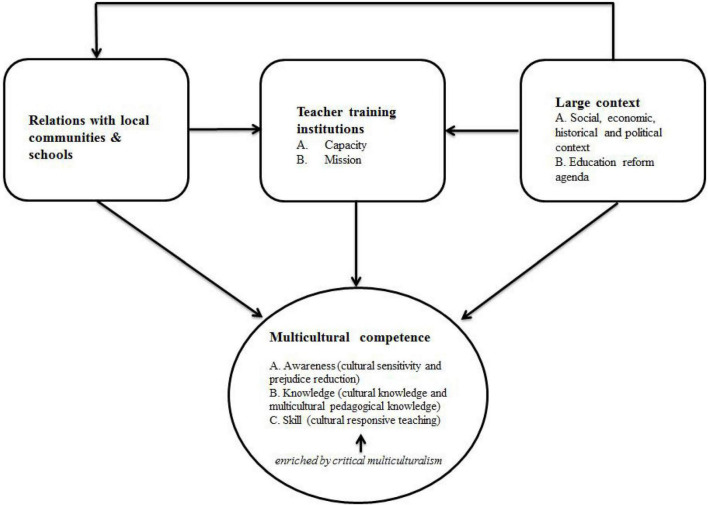
Multicultural competence training and the external forces that affect it.

Given the high degree of centralisation of educational governance in China, I slightly modify Cochran-Smith’s (2003) framework. I merge the third force, governmental/non-governmental regulations, into the larger context, as in Chinese teacher education these normally relate to political governance (Wang, 2022). The modified framework is presented in Figure 1. I use arrows in the modified framework to indicate the direction of influence. All in all, I do not change Cochran-Smith’s (2003) original meaning: in the modified framework, the *larger context* retains its influence on *external factors* and on teacher education practices.

## Methodology

The study aims to investigate how teachers’ multicultural competence is fostered by existing pre-service teacher education programmes and external influences on these programmes. The analysed data includes two syllabuses for current teacher education programmes and four interviews with teacher educators from two teacher education institutions. These are Liangxiang Teacher Education Institution (LTEI) in Chongqing municipality and Tianxiang Teacher Education Institution (TTEI) in Yunnan province which has the most complex ethnic minority demographics in China. Both LTEI and TTEI offer teacher education programmes at the bachelor level, covering all subjects offered in K–12 schools.

### Data Collection, Participants, and Methods

After obtaining the permissions from the deans of the respective Faculties of Education (Teacher Education) at LTEI and TTEI, I obtained the syllabuses of their teacher education programmes. These generally consist of the mission of the course, a curriculum framework and descriptions of a part of the curriculum, as well as a list of reference textbooks for mandatory courses.^2^

The second source of data comprises expert interviews with teacher educators. The participants (teacher educators) had provided written informed consent to participate in this study. All the four teacher educators interviewed had five or more years’ experience working and researching in teacher training and, with more than 6,000 pre-service teachers graduating from each institution annually, may be considered experts in the field. Their perspectives can be as surrogates or “crystallisation points” for teacher educators as a group, which makes data collection efficient (Bogner et al., 2009, p. 2). The similarity in interests (i.e., teacher training, ethnic minority education) between teacher educators and my own research was also considered a factor that would increase their motivation to participate in interviews and share their opinions (Bogner et al., 2009). Informed consent was elicited before each interview. Profiles of the four interviewees are given in Table 1. Based on the two theoretical frameworks presented above, the interview protocols were formulated to investigate teachers’ perceptions of the *status quo*, and further possibilities of, and obstacles to, incorporating the cultivation of multicultural competence into teacher education programmes (see Table 2).

**TABLE 1:** Interviewee profiles.

Institution	Name	Years as teacher educator	Degree and academic position	Research focus
LTEI	Lin	5–10 years	Ph.D.; Associate Professor	Teacher education
	Bai	More than 15 years	Ph.D.; Full Professor	Teacher education and ethnic minority education
TTEI	Cao	11–15 years	Ph.D.; Full Professor	Teacher education and ethnic minority education
	Ding	Less than 5 years	Ph.D.; Senior Lecturer	Teacher education and educational policy

**TABLE 2:** Interview protocol.

Question Number	Interview questions
Question 1	In your perspective, are there any differences between the competence of teachers who work in ethnic minority schools and those working in mainstream Han schools?
Question 2	In your university, is there a curriculum that provides knowledge and training for student teachers working in ethnic minority schools after their graduation? What is it?
Question 3	How is the students’ teaching practicum designed and carried out? Do students have opportunities to participate in teaching practicums in ethnic minority schools? Why?
Question 4	What kinds of internal or external factors influence whether and how teacher education institutions and teacher educators incorporate the cultivation of multicultural competence into teacher education programmes?

### Data Analysis

Content analysis was conducted on the teacher education syllabuses collected from LTEI and TTEI. Data were analysed using focus coding (Bryman, 2016). Some pre-set codes were used to sort the data, such as overall mission, curriculum structure and focus. As no searchable version of reference textbooks exists (and, collectively, they are more than 1,500 pages long), I chose to read the tables of contents first and then carefully read the section that are, explicitly or implicitly, related to multicultural competence.

The interview transcripts were analysed using the thematic analysis approach. I first read the transcripts line by line and labelled any points of interest with open codes. Then, following the thematic network approach of Attride-Stirling (2001), open codes were generated, combined and categorised to produce more abstract, organised and global codes and themes.

## Empirical Findings

The findings from this study are presented in two parts, based on the data source. I begin by discussing the content analysis of the teacher education programme syllabuses and continue with a discussion of the semi-structured expert interviews.

### Findings From the Content Analysis

Content analysis was conducted on the syllabuses of two teacher education programmes to find out how these programmes provide training in pre-service teacher multicultural competence. Based on the structures of the syllabuses, an inductive approach was used to examine mission categories and the curricula.

#### Missions: Loyalty to the Agenda of Educational Reform

The mission of a teacher education programme describes its overall aim of teacher preparation. A review of the mission statements from both institutions reveals a high degree of similarity (see Table 3). Aside from some fundamental requirements for teachers including basic knowledge, skills and theory in teaching and teaching ethics, it is evident that the New Curriculum Reform (NCR) in 2001, is the keystone of the mission statements of both programmes. LTEI’s mission statement mentions teacher education reform, but the goal is still to accommodate NCR priorities and the development of basic education. In a nutshell, the overall agenda of educational reform during the past two decades determines the contents and transformation directions of teacher education. Although the nationwide NCR is also implemented in ethnic minority education, ignorance of the needs of ethnic minorities is noticeable in both mission statements.

**TABLE 3:** Mission statements of teacher education programmes.

Institution	Mission	Comparison
LTEI	[…] Promoting reform of teacher education and adapting ***basic education development and need of new curriculum reform***, teacher education programme aims to cultivate future teachers with wholesome personalities, deep knowledge, advanced ideas and proficiency in skills. […]	Basic education curriculum reform leads the missions of the teacher education programmes in both LTEI and TTEI
TTEI	[…] master solid basic theories, basic knowledge and basic skills of pedagogy and subject teaching. Students should have good teachers’ ethics, literacy in the humanities and science as well as good mental health and wholesome personality in order to be competent teachers who are enthusiastic about a teaching career, good teaching and research to adapt to the needs of the ***new curriculum reform and development in basic education.***	

#### Curriculum Structures: Centralised, With Limited Flexibility

The syllabuses provide curriculum frameworks for both teacher education programmes, with lists of mandatory and elective courses offered to pre-service teachers.

A review of the mandatory courses reveals that though there are some differences in the two institutions, there is no course that trains teachers in multicultural competence for ethnic minority education, whether explicitly or implicitly. The mandatory courses are primarily concerned with training teachers in teaching and problem-solving. This setting of the curriculum is in complete accord with the “pedagogical knowledge and abilities” section in China’s National Teacher Education Standard (NTES) which emphasises training teachers on fundamental theories and skills in teaching, understanding students, and professional development. However, the requirements of the NTES do not include the particular needs of ethnic minority education. Indeed, the universality of mandatory courses does not refer to the challenges posed by the particularities of ethnic minority education, which are beyond universality.

Textbooks are the main reading and teaching materials in Chinese teacher education programmes. The textbooks collected for this study were used at TTEI for six mandatory courses: education laws and regulations; information and computer technology for education; classroom management; teacher accomplishment; effective teaching; and student cognition and learning. Of these, the first three focus on an introduction to the relevant laws, a specific teaching technology and classroom organisation, respectively. Though the remaining three are also inclined to provide a theoretical introduction, they each contain at least one chapter reflecting dimensions that are similar to the multicultural competence framework (see Table 4), such as recommending that teachers change their teaching strategies to adapt to their students. Students’ different characteristics (e.g., mental or cognitive) are regarded as resources that teachers need to be aware of and to respond in a way that is coherent with the propositions of the multicultural competence framework. However, these do not place a focused emphasis on the needs of ethnic minority students for their teachers to demonstrate multicultural competence, nor do they provide supporting examples or materials related to ethnic minority education.

**TABLE 4:** The points of multicultural competence reflected in teacher education textbooks.

Textbook topics (Curriculum)	Point correlated to multicultural competence framework
Teacher accomplishment	Teacher should possess basic pedagogical knowledge and consider different students’ mental characteristics to guide active student learning.
Effective teaching	Teachers should motivate student learning according to different students’ characteristics and needs.
Students’ cognition and learning	Teachers should know the cognitive characteristics of students at different levels, and their relations with learning.

The elective courses, in both cases, show a little more flexibility. For example, TTEI has made attempts to address ethnic minorities in teacher education programmes. Thus, there are some electives on subjects relevant to ethnic minorities such as “ethnic minority music,” “ethnic minority sport,” “ethnic minority psychology” and “ethnic minority education in Yunnan.” In LTEI, an elective course entitled “educational equality” has been included. One might expect this course to provide opportunities to train teachers to master the pedagogical strategies to promote educational equality in classroom teaching and management of ethnic minority education. Nonetheless, content analysis shows that this course dwells on policy issues across local and global contexts, and the universal micro-issues of teaching, rather than ethnic minority education specifically.

### Findings From Interviews

In this section, I report the relevant results from a thematic analysis of interviews with teacher educators at TTEI and LTEI. Three themes emerge from the analysis: teacher quality is a priority over teachers’ competence in dealing with diverse students; there is a divergence between teacher educators’ beliefs and practices; and a disjoint exists between teaching practicum and authentic multicultural settings.

#### Enhancing Teachers’ Basic Quality Is Given Higher Priority Than Their Competence in Dealing With Culturally Diverse Students

Two of the four teacher educators interviewed expressed their belief that enhancing teachers’ quality is a priority over developing teachers’ competence in dealing with culturally diverse students. To illustrate this viewpoint Ding, a teacher educator from TTEI, described the unbalanced distribution of qualified teachers among Han and ethnic minority areas, and urban and rural areas:

Take Yunnan province as an example; when recruiting new teachers, the schools in urban areas usually require teachers should at least have a bachelor’s degree, but the requirement declines to a College Graduation Diploma (*dazhuan*) in rural and ethnic minority areas. Indeed, there are many teachers holding only a high school diploma in some remote mountainous areas. I think this is only a way to compromise with present social reality, but as a teacher education institution, we need to develop a balance of teacher quality step by step. Promoting the equality of teachers’ basic quality is most important in current situation. (Teacher educator Ding, TTEI, 2017-6-2)

Similarly, teacher educator Bai from LTEI remarked that it is essential that educational initiatives adapt to the actual level of socioeconomic development.

Enhancing the quality of teacher teams in ethnic minority areas, such as their degrees, disciplinary knowledge and teaching skills, are urgent concerns at the present level of socioeconomic development. Indeed, in some ethnic minority areas, the number of teachers cannot be assured either. (Teacher educator Bai, LTEI, 2017-6-21)

#### The Divergence Between Teacher Educators’ Beliefs and Agency

By contrast, two other teacher educators said that they were aware of the differences in teachers’ competence in majority Han and ethnic minority areas and identified these differences in terms of ethnic minority students’ cultural characteristics and the challenge these pose to teachers’ competence.

I think the teachers for ethnic minorities and majority Han areas are somewhat different. The cultural characteristics of students in ethnic minority areas require teachers have related abilities and skills. I think it is similar to the multicultural education and related course “teaching for diverse students” that I experienced during my study visit to the United States 5 years ago. Our ethnic minorities basically have their own ethnic cultural characteristics, so I think our pre-service teachers should be taught a curriculum like “multicultural education” or a similar course at least. (Teacher educator Cao, TTEI, 2017-6-3)

It is evident that the teacher educators in this study believe that teachers’ competence in coping with culturally diverse students in ethnic minority education is of value. However, when asked about the concrete practices designed and implemented to develop this competence, it is striking that all four teacher educators indicated that these were limited. Indeed, there was limited institutional space for teacher educators’ agency, as teacher educator Lin said:

I do not think we, as teacher educators, can do too much to transform the ongoing teacher education programme. This power should be in the hand of the central education authorities. Our institution must follow the framework of national education initiatives (board policies and guidelines) to set mandatory and elective curriculums and implement teaching practice, otherwise the authorities will hold our university accountable. Therefore, autonomy is really limited for universities and our teacher educators. (Teacher educator Lin, LTEI, 2017-6-4)

Corroborating this study’s analysis of the teacher education syllabuses, teacher educators said there is some space for introducing multicultural competence in existing curriculum frameworks, such as educational equality, rural education etc. However, this is entirely dependent on the knowledge, research merit and consciousness of teacher educators to transform general content into specialised materials for ethnic minority education. Thus, for instance, teacher educators who conduct research on ethnic minority education could share their research experience with pre-service teachers in related courses, as teacher educator Cao said:

I think some of the existing courses can provide a platform to teach pre-service teachers multicultural knowledge, competence, and awareness, such as [courses on] general trends in basic education reform, or ethnic minority education in Yunnan. We can teach them related knowledge and even guide them toward ethnic minority areas for social investigation, but it depends on the experiences, consciousness and ability of teacher educators. But no policy regulates us to do that. If we want to do this in a systematic way, it really requires policy assurances from central government. (Teacher educator Cao, TTEI, 2017-6-3)

#### Disjoint Between Teaching Practicum and Multicultural Settings

The practicum is an important component of teacher education, giving pre-service teachers the opportunity to enter real classrooms for real teaching practice. LTEI and TTEI require pre-service teachers to attend teaching practicums and apply the knowledge and skills learned in their institutions to actual classroom situations for 16 and 24 weeks, respectively. When asked the possibilities of conducting teaching practicums in multi-ethnic and multicultural contexts, teacher educator Lin from LTEI said that the schools where practicums are conducted rarely have multicultural characteristics.

The teaching practicums of our college are usually carried out in Chongqing municipality and some will be in nearby districts including the ethnic minority areas in the southeast part of Chongqing. But, as you know, these areas have been assimilated by Han and the students there have few ethnic minority characteristics. (Teacher educator Lin, LTEI, 2017-6-4)

By contrast, teacher educator Ding from TTEI emphasised that Yunnan is a multi-ethnic border province and has the opportunity to implement teacher practicums in actual classrooms with students from different cultural groups. However, under the Regulation of NTES, teaching practicums have little involvement in the multicultural characteristics of ethnic minority areas.

There is a group of students choosing to do their teaching practicums in schools located in ethnic minority areas. However, [speaking] as a tutor and assessor, classroom management, lesson plan design and teaching design are prioritised [rather than multicultural competence], according to related national documents. (Teacher educator Ding, TTEI, 2017-6-2)

## Discussion and Conclusion

The content analysis shows that the mission and curriculum formulation of these two teacher education programmes do not provide enough academic preparation to enhance teachers’ competence in teaching diverse students. Instead, they focus on the universal knowledge and skills that every teacher should have. Simply put, the need for teachers’ multicultural competence in ethnic minority education is unrecognised in both teacher education programmes. The findings from the interviews with teacher educators also expose several external forces that influence the extent to which teacher education institutions can foster teachers’ multicultural competence. In this section, I will first discuss the findings from a content analysis of syllabuses to see the extent to which teacher education programmes take consideration of the particular needs of teachers providing minority education. I will then use Cochran-Smith’s framework to discuss how teacher education practices are influenced by external factors, and how both the practices and the framework are embedded within, and influenced by, the larger context (i.e., education reform agenda in China).

### Lack of Consideration of Fostering Multicultural Competence for Future Teaching

“The missions of teacher education programmes play important roles in shaping teacher candidates’ learning experiences and outcomes” (Yuan, 2018, p. 50). This study reveals that teacher competence, which is a need under the NCR, determines the mission and core content of teacher education programmes. The NCR raised the requirements for teachers in class design and teaching skills to guide explorative learning and develop problem-solving abilities. This, in turn is reflected and prioritised in teacher education programmes.

The elective courses at TTEI provide richer knowledge of ethnic minorities to pre-service teachers than the mandatory courses. In addition, a course on “ethnic minority psychology” provides a good platform for pre-service teachers to understand the culturally determined needs and characteristics of different ethnic minorities. These courses are coherent with the dimension of knowledge in the multicultural competence framework and can be further connected to teachers’ multicultural awareness and skills. However, the subordinate position and fewer class sessions given to the elective curriculum within existing programmes make this competence difficult for pre-service teachers to transform into teaching strategies (Yuan, 2018).

Overall, though the teacher education programmes in LTEI and TTEI take notice of awareness, knowledge and skills in dealing with students’ diverse characteristics, the invisibility of ethnic minority contexts or multicultural contexts in curriculums and teaching materials inevitably limits the feasibility of developing teachers’ multicultural competence for ethnic minority education.

### External Influential Forces on the Practice of Multicultural Teacher Education in China

The interviews with teacher educators verify the findings of the content analysis. The cultivation of multicultural competence for future work in ethnic minority education is, to a great extent, missing from teacher education programmes. The interviews also reveal the existence of external forces that influence teacher education practices which are coherent with the findings of Cochran-Smith (2003).

First, all four teacher educators who participated in this study identified the importance of fostering pre-service teachers’ competence in coping with diverse students in ethnic minority education. However, it was striking that they subsequently emphasised that there is limited autonomy at the lower end of the policy stream for their institutions and themselves to apply this belief to practice, and that they are constrained by the regulations laid down by the authorities (e.g., Ministry of Education). Simply put, this divergence between the beliefs and practices of teacher educators reveals that institutional capacity (including teacher educators’ own agency) to implement multicultural teacher education practices is inadequate within the current centralised governance structure for teacher education.

Since 2001, the role of the Department of Teacher Education at the Ministry of Education has gradually been transformed from being entirely government-controlled to a body that sets professional standards. Teacher education institutions have, in this process, been endowed with a certain degree of autonomy (Zhu and Han, 2006). Although governance has moved away from absolute power domination toward a certain degree of decentralisation, the power of setting professional standards for teacher education remains in the hand of the central authorities (Wang, 2022). This centralised decentralisation is consistent with this study’s finding that teacher education institutions are required to set up and implement mandatory and elective curriculums within the framework of the NTES and similar regulations. The NTES, which guides teacher education for K–12 education in China, ignores teachers’ multicultural competence in ethnic minority education. This type of governmental regulation acts as an external force that constrains the institutional capacity to facilitate pre-service teachers’ multicultural competence for ethnic minority education.

Secondly, China’s broad educational reform agenda greatly influences the missions of teacher education programmes. These missions as this study shows, articulate loyalty to education reform. Missions can undoubtedly constrain or enhance the institutional capacity of teacher education institutes in addressing cultural and diversity in their teacher education programmes (Cochran-Smith, 2003). The NCR, which has prevailed since the early 2000s, strongly emphasises student learning rather than teaching. Pre-service teacher training institutions are required to prepare teachers in accordance with the NCR’s requirements for teacher competence. As a broader reform agenda, the NCE influences how teacher education plays out in practice. Therefore, teachers’ multicultural competence, which is excluded from the reform agenda, cannot be emphasised in teacher education programmes, and institution capacity in this area is restricted.

Thirdly, interviews with teacher educators show that the locations of teaching practicums are, to a large extent, disconnected from multicultural settings. This may be related to the social context in China and the distribution of ethnic minorities within the country. Though China has 55 ethnic minorities, more than 80% live in the western border provinces and autonomous prefectures. The rest are mingled with the majority Han in the east and central provinces and have largely been assimilated by the mainstream culture. Therefore, provinces where there is a lack of communities and schools with diverse ethnic minorities face challenges in providing teacher education institutions with authentic multicultural settings for teacher practicums. Moreover, provinces where cultural diversity is lacking do not feel an urgency to foster this transformation. Therefore, if a corresponding reform in teacher education programmes is to be carried out, it must be regional, not nationwide.

Finally, the current ideology of educational equality, which derives from a recognition of the uneven socioeconomic development in China, focuses on distributive equality of teacher quality and quantity. Teaching for social justice which is extensively discussed in multicultural education scholarship requires teachers to recognise culture as foundation of learning for facilitate diverse students’ academic performance, to teach key concepts through different cultural groups’ contents and examples, to guide equal inter-ethnic communication between students from different cultural groups for eliminating discrimination (Sleeter, 2013; Cho, 2017; Darling-Hammond, 2017). However, it should be noted here that a prerequisite of social justice is distributive equality in resources and rights (Miller, 1997). In China, though ethnic minorities have been granted equal civil rights in political domains, their subordinate position in many domains, like economic development and education, has stagnated or worsened in the past four decades. As teacher educators’ perceptions have shown, the urgent concern in teacher education for ethnic minorities is to cultivate adequate and qualified teachers.

In conclusion, the capacity of Chinese teacher education institutions in fostering pre-service multicultural competence is constrained by several external forces such as educational governance and, as reflected in the locations of teaching practicums, relations with community and schools. Furthermore, what pre-service teachers’ competence covers, and the external forces that influence how teacher education plays out in practice, are influenced and somewhat determined by the larger social, economic and political context as well as the agenda for educational reform in China.

## Toward an Inclusive Teacher Education

China is still in the process of bridging the differences in developmental level between different areas and social domains, such as rural-urban or majority-minority populations. This study reveals that teacher education programmes in China lack consideration of the particular needs of teachers providing ethnic minority education, as well as how external forces and the larger context shaping teacher preparation create distance from multicultural competence. Yet, there remains a possibility—one that may be an advantage, or even a necessity—for some teacher education institutions, especially those in provinces with culturally diverse ethnic minorities, to include multicultural competence training in their teacher education programmes. Therefore, future research directions may include exploring rational ways of intervening between external forces and teacher education practices to help teacher education institutions effectively train pre-service teachers in multicultural competence, and transform their teaching to become more inclusive.

## Data Availability Statement

The original contributions presented in the study are included in the article/supplementary material, further inquiries can be directed to the corresponding author/s.

## Ethics Statement

Ethical review and approval was not required for the study on human participants in accordance with the local legislation and institutional requirements. Written informed consent to participate in this study was provided by the participants.

## Author Contributions

The author confirms being the sole contributor of this work and has approved it for publication.

## Conflict of Interest

The author declares that the research was conducted in the absence of any commercial or financial relationships that could be construed as a potential conflict of interest.

## Publisher’s Note

All claims expressed in this article are solely those of the authors and do not necessarily represent those of their affiliated organizations, or those of the publisher, the editors and the reviewers. Any product that may be evaluated in this article, or claim that may be made by its manufacturer, is not guaranteed or endorsed by the publisher.

## References

[B1] Abacioglu C. S. Volman M. Fischer A. H. (2020). ‘Teachers’ multicultural attitudes and perspective taking abilities as factors in culturally responsive teaching. *Br. J. Educ. Psychol.* 90 736–752. 10.1111/bjep.12328 31814111 PMC7496989

[B2] Attride-Stirling J. (2001). Thematic networks: an analytic tool for qualitative research. *Qual. Res.* 1 385–405. 10.1177/146879410100100307

[B3] Bai L. (2008). Duoyuan wenhua shiye zhong de jiaoshi jiaoyu [Teacher education in the perspective of multicultural eudcation]. *J. Ethn. Minor. Educ.* 19 124–128.

[B4] Banks J. A. (1993). “Approaches to multicultural curriulim reforms,” in *Multicultural Education: Issues and Perspective*, 2nd Edn. eds Banks J. A. Banks C. A. M. (Boston, MA: Allyn and Bacon), 195–214.

[B5] Banks J. A. (2014). *An Introduction to Multicultural Education*, 5th Edn. Boston, MA: Pearson.

[B6] Bogner A. Littig B. Menz W. (2009). “Introduction: expert interviews – an introduction to a new methodological debate,” in *Interviewing Experts*, eds Bogner A. Littig B. Menz W. (Hampshire: Palgrave Macmillan), 1–13. 10.1057/9780230244276_1

[B7] Bryman A. (2016). *Social Research Method*, 5th Edn. Oxford: Oxford University Press.

[B8] Cai Z. L. Kong L. X. (2014). Chedian binxiao yundong beijing xia xiangcun jiaoyu de kunjing yu chulu [Plight and solution of rural education under the moverment of merged school]. *Tsinghua J. Educ.* 35 114–119.

[B9] Cherng H. Y. S. Davis L. A. (2019). Multicultural matters: an investigation of key assumptions of multicultural education reform in teacher education. *J. Teach. Educ.* 70 219–236. 10.1177/0022487117742884

[B10] Cho H. (2017). Navigating the meanings of social justice, teaching for social justice, and multicultural education. *Int. J. Multicult. Educ.* 19 1–19. 10.1177/1745691618797957 30566379

[B11] Cochran-Smith M. (2003). The multiple meanings of multicultural teacher education: a conceptual framework. *Teach. Educ. Q.* 30 7–26.

[B12] Darling-Hammond L. (2017). Teaching for social justice: resources, relationships, and anti-racist practice. *Multicult. Perspect.* 3 133–138. 10.1080/15210960.2017.1335039

[B13] Egne R. M. (2014). Representation of the Ethiopian multicultural society in secondary teacher education curricula. *J. Teach. Educ. Sustain.* 16 54–75. 10.2478/jtes-2014-0003

[B14] Forrest J. Lean G. Dunn K. (2016). Challenging racism through schools: teacher attitudes to cultural diversity and multicultural education in Sydney, Australia. *Race Ethn. Educ.* 19 618–638. 10.1080/13613324.2015.1095170

[B15] Gay G. (2002). Preparing for culturally responsive teaching. *J. Teach. Educ.* 53 106–116. 10.1177/0022487102053002003

[B16] Gay G. (2018). *Culturally Responsive Teaching: Theory, Research and Practice.* New York, NY: Teachers College Press.

[B17] Gay G. Howard T. C. (2000). Multicultural teacher education for the 21st century. *Teach. Educ.* 36 1–16. 10.1080/08878730009555246

[B18] Gorski P. C. (2009). What we’re teaching teachers: an analysis of multicultural teacher education coursework syllabi. *Teach. Teach. Educ.* 25 309–318. 10.1016/j.tate.2008.07.008

[B19] Grant C. A. Agosto V. (2008). “Teacher capacity and social justice in teacher education,” in *Handbook of Research on Teacher Education: Enduring Questions in Changing Contexts*, eds Cochran-Smith M. Feiman-Nemser S. McIntyre D. J. Demers K. E. (New York, NY: Routledge), 175–200.

[B20] Kim Y. Choi M. (2020). Towards critical multicultural teacher education in the midst of ethno-nationalism: Korean pre-service teachers’ international learning experiences. *Teach. Teach. Educ.* 96 103–155. 10.1016/j.tate.2020.103155

[B21] Kong M. Wei R. (2019). EFL learners’ attitudes toward English-medium instruction in China: the influence of sociobiographical variables. *Linguist. Educ.* 52 44–51. 10.1016/j.linged.2019.03.005

[B22] Ladson-Billings G. (1995). Toward a theory of culturally relevant pedagogy. *Am. Educ. Res. J.* 32 465–491. 10.3102/00028312032003465

[B23] Lee M. B. (2016). Sociological perspectives on ethnic minority teachers in China: a review of the research literature. *Diaspora Indig. Minor. Educ.* 10 55–68. 10.1080/15595692.2015.1098611

[B24] May S. (2009). “Critical multiculturalism and education,” in *The Routledge International Companion to Multicultural Education*, ed. Banks J. A. (New York, NY: Routledge), 33–48.

[B25] McAllister G. Irvine J. J. (2000). Cross cultural competency and multicultural teacher education. *Rev. Educ. Res.* 70 3–24. 10.2307/1170592

[B26] Melnick S. L. Zeichner K. M. (1997). “Teacher education for cultural diversity: enhancing the capacity of teacher education institutions to address diversity issues,” in *Preparing Teachers for Cultural Diversity*, eds King J. E. Hollins E. R. (New York, NY: Teachers College Press), 23–39.

[B27] Miller D. (1997). Equality and justice. *Ratio* 10 222–237. 10.1111/1467-9329.00042

[B28] Mushi S. (2004). Multicultural competencies in teaching: a typology of classroom activities. *Intercult. Educ.* 15 179–194. 10.1080/1467598042000225032

[B29] Ni S. L. (2014). Minzu diqu jichu jiaoyu junheng fazhan yu duoyuan wenhua jiaoshi peiyang [Balanced development of basic education in ethnic minority areas and cultivation of multicultural teachers]. *Minzu Jiaoyu Yanjiu* 25 124–128.

[B30] Postiglione G. A. (2009). “The education of ethnic minority groups in China,” in *The Routledge International Companion to Multicultural Education*, ed. Banks J. A. (New York, NY: Routledge), 501–511.

[B31] Seeberg V. Minick T. (2012). Enhancing cross-cultural competence in multicultural teacher education: transformation in global learning. *Int. J. Multicult. Educ.* 14 1–22. 10.18251/ijme.v14i3.569

[B32] Sleeter C. (2013). Teaching for social justice in multicultural classrooms. *Multicult. Educ. Rev.* 5 1–19. 10.1080/2005615X.2013.11102900

[B33] Sleeter C. E. McLaren P. (1995). “Introduction: exploring connections to build a critical multiculturalism,” in *Multicultural Education, Critical Pedagogy, and the Politics of Difference*, eds Sleeter C. E. McLaren P. (New York, NY: SUNY Press), 5–32.

[B34] Te G. (2007). Duoyuan wenhua jiaoyu shiye xia de shaoshuminzu jiaoshi suzhi de chongjian [Reconstruction of ethnic minority teachers’ literacies in the perspective of multicultural education. *Minzu Jiaoyu Yanjiu* 18 17–19.

[B35] Villegas A. M. Lucas T. (2002). *Educating Culturally Responsive Teachers: A Coherent Approach.* Albany, NY: State University of New York Press.

[B36] Wang W. (2018). Researching education and ethnicity in China: a critical review of the literature between 1990 and 2014. *Front. Educ. China* 13 216–244. 10.1007/s11516-018-0012-2

[B37] Wang W. (2022). Ethnic minority cultures in Chinese schooling: manifestations, implementation pathways and teachers’ practices. *Race Ethn. Educ.* 25 110–127. 10.1080/13613324.2021.1997974

[B38] Wei R. Jiang H. Kong M. (2021). Attitudes toward trilingualism: a survey study of Chinese Mongolian university students. *J. Multiling. Multicult. Dev.* 42 291–306. 10.1080/01434632.2019.1689245

[B39] Yuan H. (2018). Educating culturally responsive Han teachers: case study of a teacher education program in China. *Int. J. Multicult. Educ.* 20 42–57. 10.18251/ijme.v20i2.1609

[B40] Zhu X. Han X. (2006). Reconstruction of the Teacher Education System in China. *Int. Educ. J.* 7 66–73.

